# Supplementary material for: Arabidopsis Root Development Regulation by the Endogenous Folate Precursor, Para-Aminobenzoic Acid, via Modulation of the Root Cell Cycle
Source: Plants (Basel). 2023 Dec 5;12(24):4076. doi: 10.3390/plants12244076 (PMC10748309; doi:10.3390/plants12244076)
Supplement: Supplementary file 1 [file plants-12-04076-s001.zip › plants-2686671-supplementary.pdf]

Figure S1

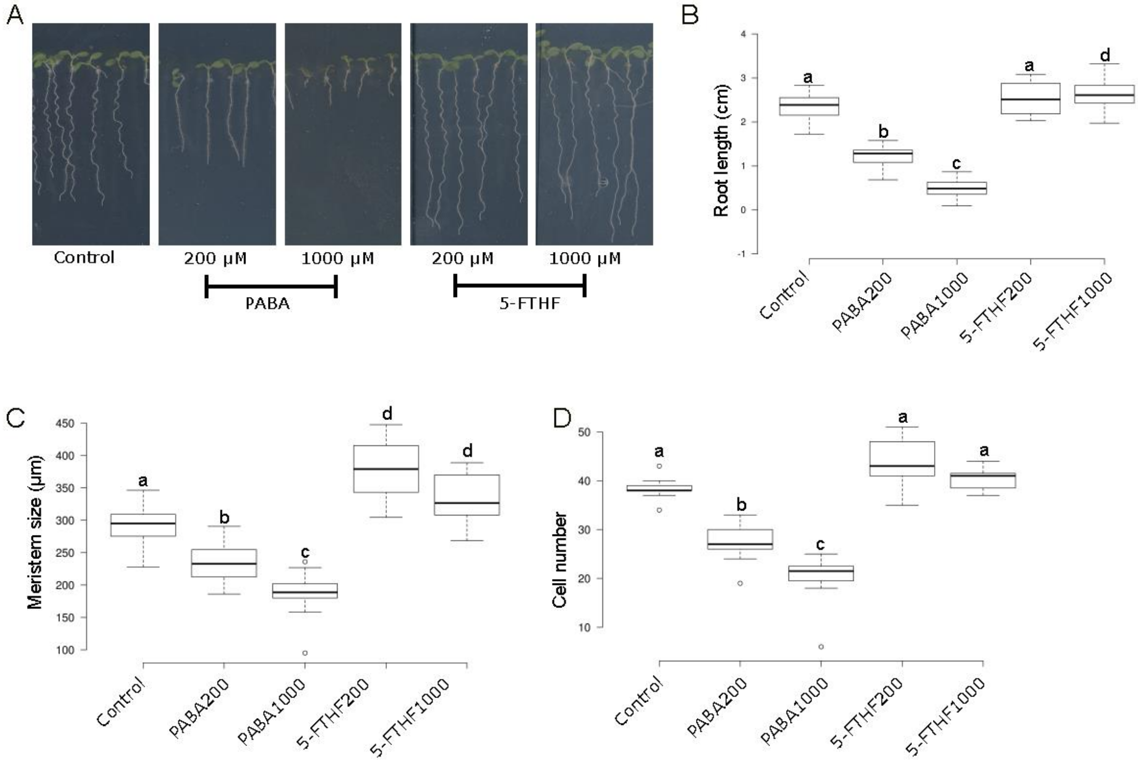

Figure S1. Comparison of the impact of PABA and 5-FTHF on the root growth. (A) The root phenotype of plants grown on PABA (200 and 1000 µM) and 5-FTHF (200 and 1000 µM). (B, C, D) Average of the mean root length, meristem size meristem, and meristematic cell number, respectively. The letters (a, b, c, d) indicate that the root length, the meristem size or cortex cell number differs from the control, as determined by one-way ANOVA with Tukey multiple testing corrections ($P < 0.05$).

Figure S2

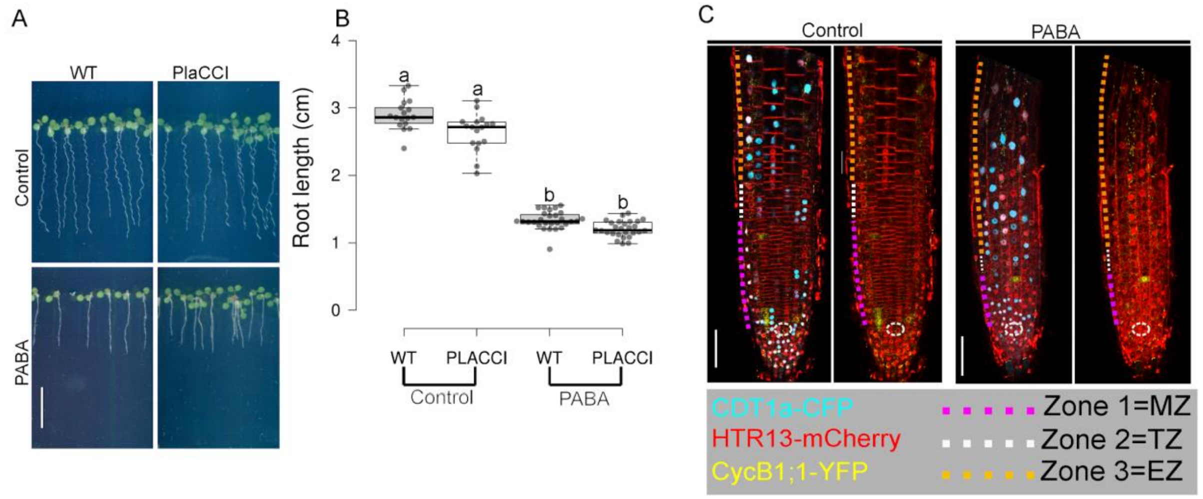

Figure S2. Use of the PlaCCI reporter line to visualize the root cell cycle state. **(A-B**), PLaCCI roots response to 200 µM PABA is similar to that of WT plants. **(A),** top panel, 6-day-old WT, and PlaCCI grown on control medium. Lower panel, WT and PlaCCI grown on PABA. **(B)** Root length of plants shown in (A). Gray and white box plots indicate WT and PLACCI plants, respectively. Letters (a, b) indicate that the root length of WT and PABA differs from the control, as determined by one-way ANOVA with Tukey multiple testing corrections ($P < 0.05$) **(C)** Confocal images displaying root tips' epidermis and cortex of 6 day-old PlaCCI plants expressing CDT1a-CFP, HTR13-mCherry, and CycB1;1-YFP constructs and grown in the absence (control) or presence of 200 µM PABA. Left panels on both control and PABA treated root display the 3 markers merged. Right panels show the same roots but without CDT1a-CFP. Dashed circle line depicts the position of the quiescent centre (QC). Dashed lines show root zone limits: Magenta (MZ, zone 1), white (TZ, zone 2) and orange (zone 3, differentiation zone). The white arrow indicates the beginning of the differentiation zone. Bar, 1 cm (A) and 20µm (C).

Figure S3

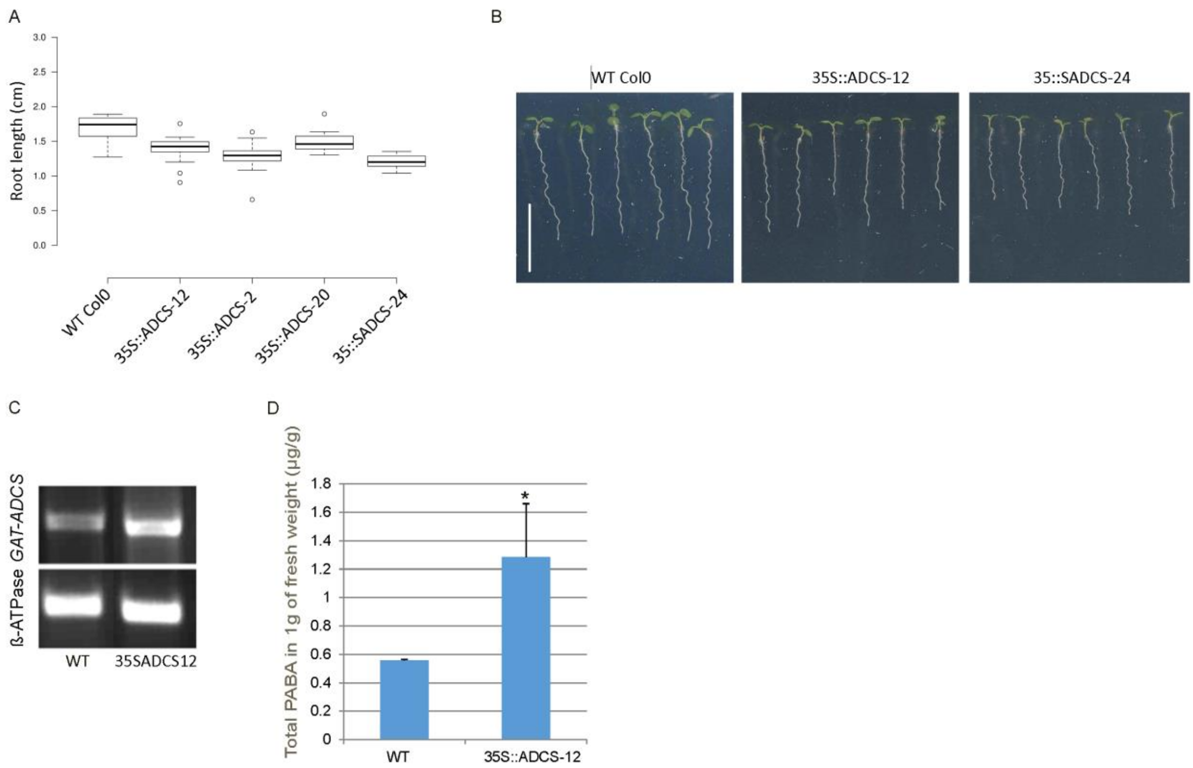

Figure S3. Characterization of *GAT-ADCS* overexpressing lines. **(A, B)** Mean root length and root phenotype of 4 *ADCS* overexpressing lines. **(C)** Confirmation of *GAT-ADCS* overexpression in 35S::*ADCS*-12 line by semi-quantitative RT-PCR. **(D)** Total PABA content in wild type (WT) and the 35S::*ADCS*-12 line. Each value is the average of three independent experiments ± standard deviation. Asterisks (*) indicate statistical significance based on the Student's T test: $P < 0.05$.
